# Changes in the Cochlear Vasculature and Vascular Endothelial Growth Factor and Its Receptors in the Aging C57 Mouse Cochlea

**DOI:** 10.1155/2013/430625

**Published:** 2013-06-27

**Authors:** David Clinkard, Hosam Amoodi, Thileep Kandasamy, Amandeep S. Grewal, Stephen Chen, Wei Qian, Joseph M. Chen, Robert V. Harrison, Vincent Y. W. Lin

**Affiliations:** ^1^Sunnybrook Health Sciences Centre, Otolaryngology/Head & Neck Surgery, Toronto, Canada M4N 3M5; ^2^Department of Otolaryngology-Head and Neck Surgery, Toronto, University of Toronto, Canada M5S 1A1; ^3^Auditory Science Laboratory, Department of Otolaryngology, Program in Neuroscience and Mental Health, The Hospital for Sick Children, Toronto, Canada; ^4^Sunnybrook Research Institute, Molecular & Cell Biology, Toronto, Canada M4N 3M5

## Abstract

*Introduction*. Previous work has shown a strong association between alterations in cochlear vasculature, aging, and the development of presbycusis. The important role of vascular endothelial growth factor (VEGF) and its receptors Flt-1 and Flk-1 in angiogenesis suggests a potential role for involvement in this process. The aim of this study was to characterize vascular structure and VEGF and its' receptors in young and old C57 Mice. *Methods*. Young (4 weeks, *n* = 14) and aged (32–36 weeks, *n* = 14) C57BL/6 mice were used. Hearing was evaluated using auditory brainstem response. Cochleas were characterized with qRT-PCR, immunohistochemistry, and gross histological quantification. *Results*. Old C57 mice demonstrated significantly decreased strial area, blood vessel number, luminal size, and luminal area normalized to strial area (vascularity). qRT-PCR showed a significant upregulation of Flt-1, a VEGF receptor, in older animals. No differences were found in VEGF-A or Flk-1. Immunohistochemistry did not show any differences in staining intensity or area with age or cochlear turn location. *Conclusion*. The marked deafness of aged C57 mice could be in part meditated by loss of vascular development and alterations in VEGF signaling.

## 1. Introduction

Presbycusis, or age related hearing loss, exerts a substantial socioeconomic impact, affecting over 25% of those 50 years old and over [1]. This loss manifests as progressive high-to-low frequency loss. Clinically, there is difficulty in speech localization and sound discrimination. The cause of presbycusis is still unclear, but hypothesized to be the result of cumulative intrinsic and extrinsic (noise and ototoxic agents) damage [2]. Cochleas affected by presbycusis demonstrate morphological alterations in the stria vascularis, hair cells, and afferent neurons suggesting a strong link between these insults and subsequent morphological alterations [3, 4].

C57BL/6 mice are a well-studied model of age related hearing loss, from age 6 months onward; these animals demonstrate progressive high-to-low frequency hearing loss with age [3, 5]. Like humans, histopathological alterations are first seen in the basal turn which progress to the apical turns as these animals first lose their outer and later inner hair cells [6]. By contrast, Swiss Webster mice do not display an age associated hearing loss or morphological alterations to their cochlea. This taken with multiple studies showing dramatic histopathological alterations to the spiral ganglion and stria vascularis in numerous models of hearing loss suggests a key role of the vascular network in the maintenance of hearing [7, 8]. 

Vascular endothelial growth factor (VEGF) and its two major receptors Flt and Flk have a critical role in angiogenesis and the maintenance of tissue vascularization [9, 10]. Soluble VEGF interacting with the tyrosine kinase receptor Flk is responsible for most of the aforementioned effects. The role of Flt is still unclear; it is known to exist in two forms, soluble and membrane bound, and is hypothesized to have a role in sequestering VEGF and helping to spatially direct vessel formation [11]. 

The role of VEGF in the cochlear is still unclear. VEGF is expressed in the normal cochlea and is upregulated in response to hypoxia, oxidative stress, and decreased in response to aging [8]. Previous work in our lab on the normal hearing Swiss Webster did not show any change in VEGF expression with age [12].

The aim of this research was to determine if aging is associated with alterations in VEGF expression and vascular structure in C57BL/6 mice and how these compare to normal hearing SW animals of the same age. Both qualitative and quantitative assessment of VEGF and its receptors were carried out with immunohistochemistry and quantitative qRT-PCR to investigate this hypothesis.

## 2. Methods

### 2.1. Animal Models

C57BL/6 mice were obtained from Charles River Laboratories (Montreal, QC) and allowed a one-week acclimatization period before experimentation began. Fourteen young (4 week old) and 14 old (retired breeders 32–36 weeks old) were used. Animals had *adlib* access to water and food and were kept on standard 12 h light/dark cycles at 23°C. All experiments were performed with the approval of the University of Toronto Animal Care Committee and the Canadian Standards of Ethical Treatment of Laboratory Animals.

### 2.2. Auditory Brainstem Responses

Auditory brain stem responses (ABRs) were performed in a sound-attenuating chamber on all the lightly anesthetized (ketamine 15 mg/kg and xylazine 2.5 mg/kg) young and old animals. 

 ABRs were recorded using skin electrodes in a standard vertex to postaural configuration. Acoustic stimuli were short (1 msec rise/fall, 2 msec plateau) tone pips of 4, 8, 16, and 32 kHz presented between 70 dB peSPL and −20 dB peSPL. Potentials were band-pass filtered (150 Hz to 3 kHz) and amplified conventionally. After A-D conversion and artifact rejection, signals were averaged (Cambridge Electronic Design 1401 intelligent interface with 80286 host). In general, 300 averages of a 25 msec window were used.

After hearing status was assessed, young animals were randomly allocated into 2 groups. Group 1 was immediately sacrificed via cervical dislocation and cochlea isolated in Dulbecco's Modified Eagle Medium (DMEM) (Sigma, Oakville, ON) with 1% FBS (Sigma, Oakville, ON) for immunohistochemistry (*n* = 4) or qRT-PCR (*n* = 6). Group 2 animals (*n* = 4) were injected with Fluorescein isothiocyanate (FITC) conjugated lectin (0.1 mL/g Sigma, Oakville, ON) via femoral vein injection, allowed to rest for 5 minutes under a heat lamp, and sacrificed, and the cochlea isolated for immunohistochemistry. This process was then repeated for the older animals.

### 2.3. Immunohistochemistry

Cochleas were cleaned of connective tissue and the stapes removed, and a small fenestration was made in the apical turn. Cochleas were then fixed in 4% paraformadehyde for 30 minutes. Following fixation, cochleas were decalcified in 10% Ethylenediaminetetraacetic acid (EDTA) (Sigma, Oakville, ON) for 48 hours. Following decalcification, cochleas were placed in an increasing sucrose gradient (10, 30, 50%) for 24 hours each. Tissue was then embedded in optimal cutting temperature compound (OCT) (Tissue-Tek, Sakura, Netherlands), frozen, and sectioned (10 *μ*m) onto charged sides.

Primary antibodies VEGF-A, Flt-1, and Flk-1 (Santa Cruz biotechnology, Santa Cruz, Ca) were made 1 : 300 in 10% normal goat serum (NGS) (Gibco, Carlsbad, CA), 0.05% Triton-X (Sigma, Oakville, ON) in PBS, and slides incubated for 12 h at 4°C on a nutuator. Anti-goat cy3 secondary antibody (Jackson Laboratories, West Grove, PA) was diluted 1 : 500 in 10% NGS, 0.05% Triton-X for 4 hrs at room temperature on a nutator. A phalloidin-FITC (Sigma-Aldrich, Oakville, ON) counterstain (1 : 500) was applied for 15 minutes prior to mounting with Vectashield (Vector Laboratories, CA). Images were taken using a Zeiss LSM 510 confocal using the 60x water immersion lens. Images were then further processed using ImageJ v1.46 (NIH).

### 2.4. Vascular Structure Quantification

Cochleas from Group 2 were prepared and mounted as previously described. Images were taken using a Zeiss LSM 510 confocal using the 60x water immersion lens. ImageJ v1.46 (NIH) was used to quantify lumen area, vessel number, and strial area by two trained and blinded reviewers. When substantial disagreement was present (>5%) a third reviewer was utilized. 

### 2.5. qRT-PCR

After cochleas were cleaned of connective tissue, they were transferred to RNAlater (Qiagen, Valencia, CA) and dissection carried out to isolate the apical and basal turn. Three cochlear turns were pooled per sample. Tissue was homogenized and RNA extracted using an RNeasy kit (Qaigen, Valencia, CA) according to manufactures protocol. RNA purity was then assessed on a NanoVue 4282 Spectrophotometer (GE Healthcare). Samples with a UV260/280 >2.0 and <1.8 were repurified or discarded. 

cDNA synthesis was performed using SuperScript II cDNA synthesis kit (Invitrogen, Burlington, On) using 0.25 *μ*g total RNA according to manufactures protocol. 

qRT-PCR was performed in triplicate using SYBR Green Supermix (Bio-Rad, CA, USA) in a StepOne PCR Detection System (Invitrogen, Burlington, On). 

The following primers were used: GAPDH, VEGF-A, Flt-1, and Flk-1. (Integrated DNA Technologies, CA, USA). 1 mL of cDNA, 0.5 mL of 5000 nM forward and reverse primers, 10.5 mL RNAase free water (Bio-Rad, CA, USA), and 12.5 mL of SYBR Green Supermix (Bio-Rad, CA, USA) were combined for a total reaction volume of 25 mL. Reactions were run in triplicate and amplification products were detected in a StepOne Real-Time PCR detection system (Bio-Rad, CA, USA). Primers were as previously described [12].

The 2^−∆∆*Ct*^ method was used to assess for relative changes of mRNA levels [13]. Values were normalized with GAPDH and the young C57 apical turns. 

### 2.6. Statistics

Unpaired *t*-tests were used to compare auditory brainstem responses, PCR gene expression levels, vascular area, vessel number, and strial vascularity. Microsoft Excel (Microsoft, Seattle, WA) was used for data analysis. A *P* of <0.05 was determined to be significant.

## 3. Results

Where relevant, data for Swiss Webster Mice is presented from previously published experiments in this experiment series for interspecies comparison [12].

### 3.1. Auditory Brainstem Responses

**Figure 1:**
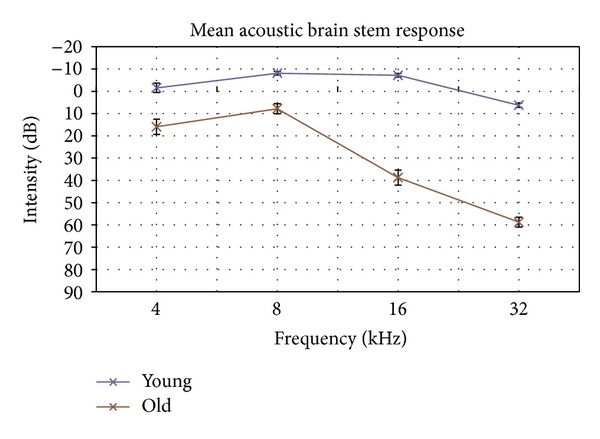
ABR demonstrated significant attenuation of response at all pure tone frequencies confirming elevated thresholds in old C57 mice.

The mean thresholds were −1.56, −8.125, −7.19, and 6.25 dP peSPL at 4, 8, 16, and 32 pure tone stimuli, respectively, in the younger C57 mice. The older C57 mice had thresholds of 15.94, 7.815, 38.75, and 58.75 dP peSPL at 4, 8, 16, and 32 pure tone stimuli (Figure 1). There were significant differences in thresholds at all frequencies between the young and old animals (*P* < 0.05). The average hearing loss across all frequencies was 32.9 dB.

### 3.2. qRT-PCR

There was no significant difference in VEGF, Flt-1, or Flk-1 gene expression between the apical or basal turns in young and old mice. There was no significant difference in expression in turn expression between young and old mice. 

**Figure 2:**
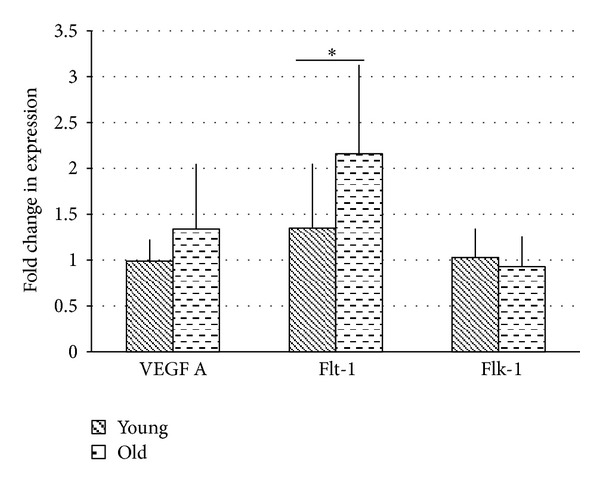
There was a significant upregulation in Flt-1 in older animals (2.16-fold versus 1.36-fold, *P* < 0.05). No difference was seen in VEGF-A or Flk-1 expression.

When turn results were pooled to examine total cochlear expression, there was a significant difference in Flt-1 expression (*P* = 0.02) between young and old mice. No significant differences were present in VEGF or Flk-1 expression between young and old mice (Figure 2).

### 3.3. Immunohistochemistry

**Figure 3:**
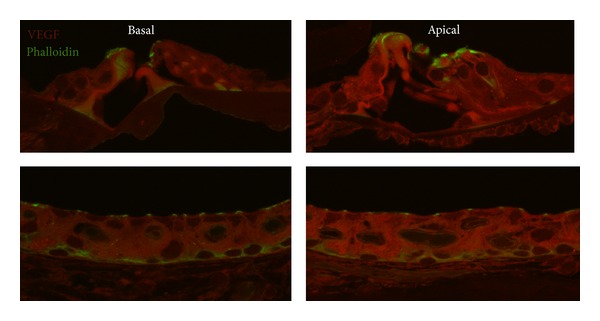
There was no basal to apex difference in VEGF labelling intensity in old C57 mice.

VEGF-A, Flt-1, and Flk-1 labeling was detected in the strial vascularis, the Organ of Corti, and spiral ganglia. There were no significant changes in any labeling or in labeling intensity from base to apex in either the young or old C57BL/6 mice. Furthermore, there was no significant difference in overall labeling when comparing the base of young versus old C57BL/6 mice. There was also no significant difference in overall labeling when comparing the apex of young versus old C57BL/6 mice (Figure 3). 

### 3.4. Gross Vascular Structure

C57 mice showed substantial differences in strial area, total luminal area as a percentage of strial area, and blood vessel number. Older animals displayed a significantly decreased area of the strial vascularis when compared to younger animals (3391.6 ± 926 *μ*m^2^ versus 4220.8 ± 1053 *μ*m^2^, *P* < 0.05). There was a significant decrease in basal to apical strial area (28%); this was not affected by age.

**Figure 4:**
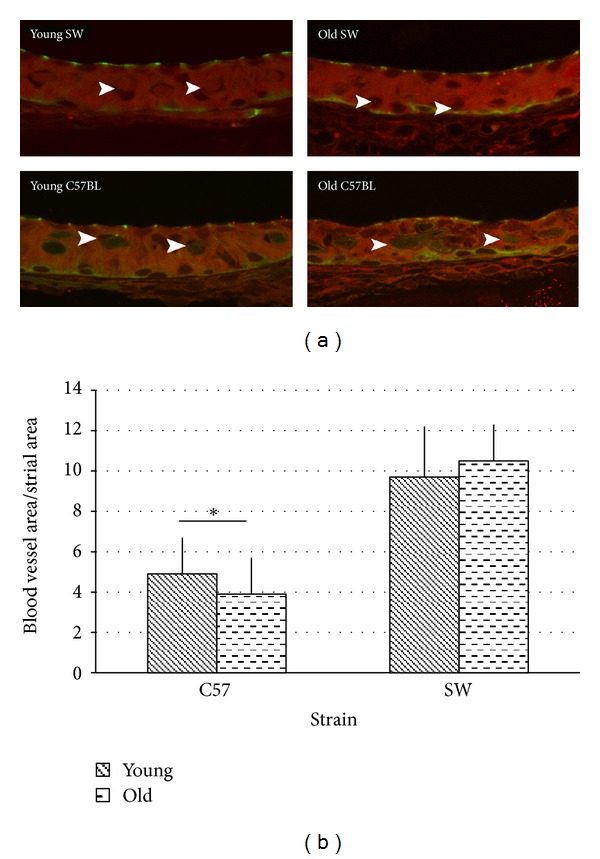
(a) Old C57BL mice display marked decreases in vessel number and size. Old C57 striae appear significantly more disorganized than young C57 animals. (b) Vascularity decreased significantly with age in C57 animals (4.9% ± 0.02% versus 3.9% ± 0.02%,  *P* < 0.05). There was no difference in vascularity with age in young versus old SW animals (9.7% ± 0.02% versus 10.5% ± 0.02%).

Normalizing for strial area, the area occupied by blood vessels was significantly decreased in older animals as compared to younger animals (3.9% versus 4.9% *P* < 0.05) (Figures 4(a) and 4(b)). There were no differences in vascularity as percentage of strial area between the apex and basal turns in both young and old animals. 

**Figure 5:**
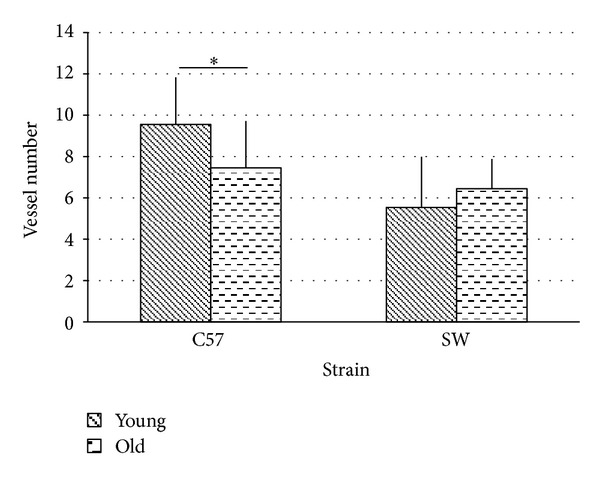
There was a significant decrease in vessel number (7.4 versus 9.56, *P* < 0.05) in aged C57 mice compared to young animals. There was no significant difference due to age (young 5.5, old 6.4) in vessel number in the SW animals. SW animals had a significantly reduced number of vessels compared to C57 at all ages (*P* < 0.05).

Older animals displayed a significantly reduced number of blood vessels when compared to younger animals (7.45 versus 9.56, *P* < 0.05) (Figure 5). There was significant apex to basal differences in both young (8.0 versus 5.45, *P* < 0.05) and old animals in vessel number (7.02 versus 4.47, *P* < 0.05).

**Figure 6:**
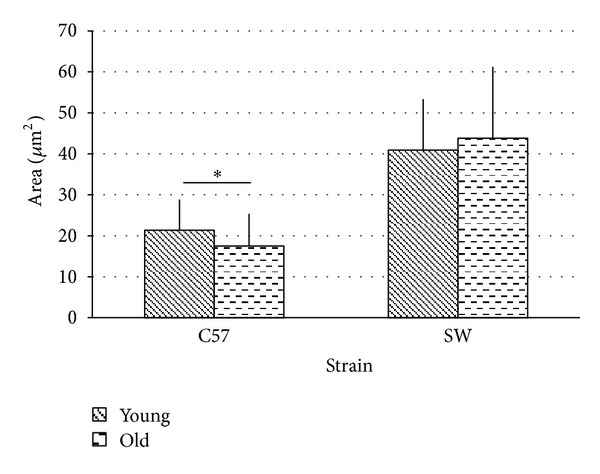
Vessel area displayed a trend toward decreased luminal area (17.5 *μ*m^2^ versus 21 *μ*m^2^, *P* = 0.052) in old C57 mice. There was no difference in vessel area due to age in SW animals (young 40.93 *μ*m^2^, old 43.85 *μ*m^2^). C57 animals had a significantly reduced area compared to SW.

There was a trend towards older animals having a decreased average luminal area as compared to young animals (17.42 *μ*m^2^ versus 21.35 *μ*m^2^, *P* = 0.052) (Figure 6). Older animals had a significantly increased apical lumen size when compared to young animals (23.2 *μ*m^2^ versus 16.51 *μ*m^2^, *P* < 0.05). No difference was observed in the lumen area of the basal turn between old and young animals. 

## 4. Discussion

Angiogenesis is a complex process mediated by a series of ligands in spatial and temporally specific manner. Numerous factors have been implicated: TGF-*α*, TGF-*β*, hepatocyte growth factor, acid fibroblast growth factor, the interleukins and VEGF. VEGF and its receptors Flt-1 and Flk-1 appear to play a rate-limiting role in this process. VEGF is regulated by hypoxia, as well as numerous oncogenes and growth factors [10, 11]. 

The link between vascularity and hearing has long been suspected, and the current work provides further support for this hypothesis [14]. Aging was associated with substantial gross morphological differences between older and younger C57 mice. 

Younger animals had a significantly larger absolute strial area as compared to the older animals. This was an unexpected finding given that the physical size of a young cochlea is substantially smaller than a cochlea harvested from an older animal. As would be expected given the larger strial area the number and size of blood vessels were also significantly increased. However, when the area occupied by blood vessels was normalized using strial area, older animals had a significantly reduced area of blood vessels to strial area, suggesting that blood vessels are lost with age.

Previous work in our laboratory demonstrated normal hearing young and old SW mice have no age related difference in vascularity, gross morphological structure, or VEGF expression as evaluated by immunohistochemistry and qRT-PCR [12]. When interspecies comparisons are made, young and old SW mice had an absolute strial area that 41% and 33% (resp.) matched young and old C57BL/6 animals. SW animals did not display the striking decrease in absolute strial area that was apparent in the C57BL/6 animals with age (Figure 4(b)). 

When the area of individual blood vessels is examined, SW animals had 104% and 133% greater luminal areas than young and old C57BL/6 animals (Figure 5). SW animals displayed a slight increase in vessel number with age (5.54 versus 6.54, n.s) though they had absolute numbers that were lower than matched C57 mice. Normalizing for strial area, the area of blood vessels in SW animals was 114% and 150% greater in young and old animals compared to matched C57 animals (Figure 3(b)). 

The current investigation failed to find increased expression of VEGF or its receptors via immunohistochemistry, though qPCR did show a significant upregulation of Flt-1. Previous investigations with Western blots reported significantly increased VEGF labeling with age [8]. This difference could be due to differences in experimental protocol such as utilizing qPCR for protein quantification as opposed to western blotting techniques. Alternatively, recent research suggests that Flt-1 can undertake a scavenging function by binding VEGF. Should this be the case, the increased Flt-1 expression would decrease the apparent expression of VEGF [11].

The regulation of VEGF and angiogenesis is complex. It has long been known that cochlear hypoxia due to reductions of cochlear blood flow occurs with age [15, 16] and that temporary hypoxia can result in reversible hearing loss [17]. Recent work has demonstrated up-regulation of VEGF occurs in response to cochlear hypoxia and as previously noted, with age [8, 9, 18].

These findings suggest a possible mechanism between decreased blood flow and VEGF expression. Our working hypothesis suggests an endogenous insult of the strial vascularis, that ultimately decreases vasculogenesis. With time, a hypoxic environment develops as flow is decreased, stimulating the expression of multiple hypoxic genes such as HIF-alpha. HIF-alpha stimulates the expression of VEGF and, in susceptible cells such as the hair cells responsible for high frequency sensation, activates the apoptotic pathways. VEGF up-regulation is unable to increase the vascularity, and the process is potentiated. 

This framework raises the intriguing possibility of early pharmaceutical intervention: could early delivery of VEGF preempt the development of a hypoxic environment? Currently there are no pharmacologic compounds available that alter the development of presbycusis. Not surprisingly, increased VEGF expression has been heavily implicated in the pathological angiogenesis associated with cancer, and a significant research has focused on developing pharmaceutical interventions such as bevacizumab or razumab to inhibit angiogenesis [19, 20]. In addition, local delivery of VEGF is currently being investigated as a possible therapy for post-MI hearts or postischemic neurovascular remodeling [21, 22]. 

A major thrust of this research was to determine the involvement of the VEGF signaling pathway in presbycusis and if commercially available pharmaceuticals would have a potential role in preventing presbycusis development. Significant further work is needed to determine the time course of vascular alterations and address the significant technical limitations associated with delivery of VEGF to the cochlea. 

## 5. Conclusion

In summary, Immunohistochemistry and qPCR were both used to examine gene expression. No differences were seen with immunohistochemistry. qPCR showed a significant up-regulation of Flt-1 with age, suggesting a potential involvement of Flt-1 in hearing loss.

We demonstrate that C57BL/6 animals exhibit hearing loss and an associated decrease in strial vascularity with age. These changes are not apparent in SW mice of similar ages, a normal hearing mouse model [12]. 

## References

[1] Parham K, McKinnon BJ, Eibling D, Gates GA (2011). Challenges and opportunities in presbycusis. *Otolaryngology—Head and Neck Surgery*.

[2] Gates GA, Mills JH (2005). Presbycusis. *The Lancet*.

[3] Di Girolamo S, Quaranta N, Picciotti P, Torsello A, Wolf F (2001). Age-related histopathological changes of the stria vascularis: an experimental model. *Audiology*.

[4] Gratton MA, Schmiedt RA, Schulte BA (1996). Age-related decreases in endocochlear potential are associated with vascular abnormalities in the stria vascularis. *Hearing research*.

[5] Li H-S, Borg E (1991). Age-related loss of auditory sensitivity in two mouse genotypes. *Acta Oto-Laryngologica*.

[6] Willott JF (1986). Effects of aging, hearing loss, and anatomical location on thresholds of inferior colliculus neurons in C57BL/6 and CBA mice. *Journal of Neurophysiology*.

[7] Hequembourg S, Liberman MC (2001). Spiral ligament pathology: a major aspect of age-related cochlear degeneration in C57BL/6 mice. *Journal of the Association for Research in Otolaryngology*.

[8] Picciotti P, Torsello A, Wolf FI, Paludetti G, Gaetani E, Pola R (2004). Age-dependent modifications of expression level of VEGF and its receptors in the inner ear. *Experimental Gerontology*.

[9] Picciotti PM, Fetoni AR, Paludetti G (2006). Vascular endothelial growth factor (VEGF) expression in noise-induced hearing loss. *Hearing Research*.

[10] Ferrara N, Gerber H-P, LeCouter J (2003). The biology of VEGF and its receptors. *Nature Medicine*.

[11] Kappas NC, Zeng G, Chappell JC (2008). The VEGF receptor Flt-1 spatially modulates Flk-1 signaling and blood vessel branching. *Journal of Cell Biology*.

[12] Kandasamy T, Clinkard D, Qian W (2012). Changes in expression of vascular endothelial growth factor and its receptors in the aging mouse cochlea, part 1: the normal-hearing mouse. *Journal of Otolaryngology—Head and Neck Surgery*.

[13] Schmittgen TD, Livak KJ (2008). Analyzing real-time PCR data by the comparative CT method. *Nature Protocols*.

[14] Schuknecht HF (1955). Presbycusis. *Transactions of the American Laryngological, Rhinological and Otological Society*.

[15] Prazma J, Carrasco VN, Butler B, Waters G, Anderson T, Pillsbury HC (1990). Cochlear microcirculation in young and old gerbils. *Archives of Otolaryngology—Head and Neck Surgery*.

[16] Harrison RV (1998). An animal model of auditory neuropathy. *Ear & Hearing*.

[17] Attias J, Sohmer H, Gold S, Haran I, Shahar A (1990). Noise and hypoxia induced temporary threshold shifts in rats studied by ABR. *Hearing Research*.

[18] Donadieu E, Riva C (2012). Hypoxia-inducible factor 1-alpha target protein up-regulation in Hypoxic cochlear neurons is associate with aged-related hearing loss in C57BL/6 mice. *Ageing Research*.

[19] Ferrara N, Hillan KJ, Gerber H-P, Novotny W (2004). Discovery and development of bevacizumab, an anti-VEGF antibody for treating cancer. *Nature Reviews Drug Discovery*.

[20] Willett CG, Boucher Y, di Tomaso E (2004). Direct evidence that the VEGF-specific antibody bevacizumab has antivascular effects in human rectal cancer. *Nature Medicine*.

[21] Lee RJ, Springer ML, Blanco-Bose WE, Shaw R, Ursell PC, Blau HM (2000). VEGF gene delivery to myocardium: deleterious effects of unregulated expression. *Circulation*.

[22] Hermann DM, Zechariah A (2009). Implications of vascular endothelial growth factor for postischemic neurovascular remodeling. *Journal of Cerebral Blood Flow and Metabolism*.

